# Patient centered medication treatment for opioid use disorder in rural Vermont: a qualitative study

**DOI:** 10.1186/s13722-024-00529-8

**Published:** 2025-01-14

**Authors:** Emily G. Hichborn, Owen B. Murray, Eilis I. Murphy, Tess E. Gallant, Sarah K. Moore, Bethany M. McLeman, John Saroyan, Anthony Folland, Megan Mitchell, Lisa A. Marsch

**Affiliations:** 1 Center for Technology and Behavioral Health, Geisel School of Medicine, Dartmouth College, Lebanon, NH 03766 USA; 2 Vermont Blueprint for Health, Vermont Agency of Human Services, Waterbury, VT 05671 USA; 3 3Vermont Department of Health, Division of Substance Use Programs, Burlington, VT 05401 USA

**Keywords:** Opioid use disorder, Medication treatment, Patient perspective, Qualitative, Patient-centered care

## Abstract

**Background:**

Opioid-related fatal overdoses are occurring at historically high levels and increasing each year. Accessible social and financial support are imperative to the initiation and success of treatment for Opioid Use Disorder (OUD). Medications for Opioid Use Disorder (MOUD) offer effective treatment but there are many more people with untreated OUD than receiving evidence-based medication. Patient-centered care is associated with increased care utilization for substance use disorders. This qualitative study explored the patient perspective of OUD care through a Patient-Centered Care (PCC) framework to illuminate patients’ sense of engagement in care.

**Methods:**

Fifteen semi-structured telephone interviews were conducted from August through November of 2021 regarding patient experiences receiving MOUD in 13 Vermont Hub and Spoke clinics. Emergent themes were deductively mapped to PCC domains of Therapeutic Alliance, Individualized Care, Shared Decision-Making, and Holistic Care.

**Results:**

Participants indicated that PCC fostered engagement and often characterized MOUD clinics they no longer attended as lacking in PCC. Themes related to Therapeutic Alliance were the most prevalent and suggest pathways to retention. Individualizing care through flexible appointment scheduling was strongly valued, while inflexible scheduling fostered fear of not getting medication. Some participants indicated they were less likely to remain in care when providers did not include them in decisions about medication type, dose, or formulation. Participants also appreciated holistic biopsychosocial care and care referrals.

**Conclusions:**

Patient-centered MOUD care was important to participants and encouraged engagement in care. Prioritizing alliance with patients, adapting care to patient needs and preferences particularly when scheduling, including patients in medication decisions, and biopsychosocial attention to patients are congruent with patient perception of desirable MOUD care. Having this understanding of an established, leading MOUD treatment system may serve to benefit states looking to implement this model, or for states who are looking to improve the model they already have in place, potentially leading to higher treatment and retention rates.

**Trial registration:**

This was not a clinical trial involving an intervention, and therefore registration was not required.

## Background

Opioid related overdoses in the US are a national epidemic [1] and are increasing in rural areas in Vermont, California, Connecticut, Maryland, New York, North Dakota, North Carolina, and Virginia [2]. Medications for Opioid Use Disorder (MOUD) - methadone, buprenorphine, and naltrexone - have strong evidence for effectiveness [3]. While the availability of MOUD has increased, 81.7% of people with opioid use disorder (OUD) aged 12 and older in 2022 had not received treatment in the past year [4]. Rural areas have particularly struggled with fewer treatment options and a growing number of patients in need of services [5–7]. The Vermont Department of Health reported a 500% increase in drug overdose related deaths with at least 79% involving one or more opioids between 2010 and 2022. In 2022, it was reported that 91% of those deaths involved at least one opioid [8]. Additionally, from 2017 to 2019 the number of deaths related to heroin and fentanyl decreased in three urban counties, and increased in almost all rural counties [9, 10]. Moreover, there are 16 states where the urban and rural rates were increasingly similar, highlighting the need to address substance use in these rural areas. The CDC reported in 2020 that the rate of overdose deaths in the US involving synthetic opioids (non-methadone) occurred most frequently compared to other drugs, while deaths related to natural and semisynthetic opioids were nearly 13% higher in rural counties than urban counties [2]. With such high prevalence rates, building a system of care for OUD has been a challenge nationwide, particularly in rural settings.

To help combat these challenges, Vermont initiated evidence-based MOUD in the early 2000s [11], but access to buprenorphine in the state remained limited into the next decade [12]. In response, Vermont designated OUD as a chronic condition and created the Vermont Care Alliance for Opioid Addiction in 2013, which today is known as the Vermont Hub and Spoke Model System [11]. In this system of care, Opioid Treatment Programs (OTPs) serve as “Hubs” for high-need patients with OUD while OBOTs at primary care and specialty clinics serve as “Spokes.” Patients can transfer between these two outpatient levels of care based on their needs. This model has made Vermont a national leader in OUD care [11], with the highest MOUD treatment capacity in the US [4].

Hub and Spoke models where OTPs serve as Hubs have also been implemented in California [13] and Pennsylvania [14], and modified versions of the model have been implemented in Washington [15], Tennessee [16, 17], and a New York State Veterans Administration Medical Center [18]. With a growing number of states beginning to replicate the Hub and Spoke system and the continued need to increase service access, particularly in rural areas, it is important to understand which components of care are important to patients. Patient-centered care prioritizes patient-specific needs and strives to improve the equality, both in power and responsibility, and collaborative nature of patient-provider dynamics. As such, it has been associated with positive treatment outcomes [19] including increased retention [20, 21], which has been demonstrated to reduce mortality and morbidity [3]. Only one other study has evaluated the patients’ perspectives of the Hub and Spoke system [22], with another in process [23]. We sought to explore which patient-centered care principles were noted by patients in Vermont’s recognized MOUD care system, which factors were missing, and which components could be improved upon from the patient perspective so other Hub and Spoke models can promote patient-centered care.

## Methods

### Design

We conducted a cross-sectional qualitative study of patients engaged in rural MOUD care in Vermont between August and November of 2021. An interview guide was developed to elicit patient experiences receiving MOUD in Vermont. The Patient-Centered Care (PCC) framework employs a biopsychosocial, holistic approach to patient-centered care and can be applied to different providers, treatments, and settings to explore the care that patients receive [19]. This PCC framework has four core domains: (1) Holistic Care - “wraparound services that meet clients’ needs at a given point in time” operationalized at addiction treatment settings as integrating or coordinating physical health, mental health, and psychosocial services; (2) Individualized Care - “health care providers’ efforts to understand clients’ unique needs, preferences, and expectations”; (3) Shared Decision Making - “the client and provider engaged in dialogue to reach a mutual decision on the best course of treatment including choice of the intervention, its frequency, duration, and follow-up plans”; and (4) Therapeutic Alliance - “relationships that were non-judgmental, respectful, accepting and/or empathic, understanding, and warm and kind” [19]. The PCC framework was used to provide structure to the organization of codes that were identified through preliminary analysis. The study was approved by the Institutional Review Board (IRB) of the Dartmouth College Committee for the Protection of Human Subjects.

### Participant recruitment

This project utilized a convenience sample of individuals receiving care at a Vermont Hub or Spoke, aged 18 years and older, English speaking, and had received their most recent dose of MOUD within the last 45 days. Potential participants were recruited through the posting of informational flyers at Vermont Hub and Spoke clinics statewide, in community areas such as town bulletin boards and local health centers. Flyers were also widely distributed to local partners via email and posted on web-based community platforms (e.g., Craigslist.com and social media accounts) to engage patients across the spectrum of care in Vermont – including those not in treatment. Interested parties contacted the research team via phone or email to express interest, learn more about the research study, and screen for eligibility. If patients were eligible and interested in completing the interview, the research team member reviewed and obtained verbal consent and conducted a brief demographic screening and the semi-structured interview.

### Demographics and interviews

Once enrolled, participants were asked general questions about their sex, race, gender, ethnicity, and treatment history. Answers were documented on paper and uploaded to Excel. No identifying information was collected. Research staff, neither of whom were directly involved with implementation at the clinics, were trained in qualitative interviewing methods prior to independently conducting 30-60-minute semi-structured interviews by telephone. All participants were compensated with a $100 electronic gift card for their time. We intended to recruit approximately 12 Vermonters located throughout the state, aiming for geographical spread over the Hub and Spoke coverage area, consistent with suggested interview saturation in homogeneous study populations [24]. Interviews were conducted without field notes and were recorded and transcribed verbatim using Rev.com transcription services.

### Analysis

Demographics were uploaded to Excel and analysed descriptively using frequencies and mean. Transcribed interviews were uploaded to qualitative coding software (Atlas.ti, v8.4) [25] within one week of their completion. Two interviewers independently reviewed and de-identified transcripts and used directed content analysis [26, 27] based on the interview questions to code patterns and divergent perspectives surrounding patient experiences of rural MOUD care. Reviewers met weekly to enhance confirmability of findings and achieve inter-rater reliability, and reviewed findings with the other authors.

Deductive analyses utilized the PCC framework domains to facilitate organization of interview codes, but analysts remained open to emergent content. Following initial coding, the PCC framework was used to organize the codes into themes that mapped onto PCC domains or fell outside the framework [19]. All decisions regarding this mapping were made in consensus by the team, who mapped data to PCC framework domains and their defining characteristics. Following the framework, the emotional valence and frequency of each theme was determined across participants. Valence was represented by the symbols: “+”, “–”, or “O”. This categorization demonstrates a consistent relationship between positively reported themes (+) and the embodiment of PCC principles in the respective care settings of all patients discussing those themes. Conversely, negatively reported themes (-) signal a unanimous perception among reporters that their care lacked PCC. Instances of mixed reports (O) was noted when opinions on a specific theme varied between participants, encompassing both positive and negative perspectives. The frequency of themes was categorized into three groups: majority (themes appearing in more than 7 interviews), some (themes present in 2–7 of the interviews), and minimal (themes discussed in 1–2 interviews). This categorization is based on the coding frequency of relevant text segments.

## Results

### Participant demographics and characteristics

Fifteen patient participants were recruited and enrolled in this project. Nine of the 15 participants identified as female, all identified as White, 7 participants reported being employed, and none reported being unhoused. All 15 participants considered themselves in active OUD care at one of 13 clinics located in five Vermont counties (1–3 participants per county). Four of these counties (80%) meet HRSA designation for rurality [28]. Nine (60%) participants were receiving buprenorphine, 6 (40%) were receiving methadone, and zero were receiving naltrexone. Additionally, 9 participants (60%) reported current MOUD treatment for more than a year and 12 participants (80%) had previously received MOUD at other Vermont treatment locations.

### PCC themes

Consistent with findings of Marchand, et al. [19], some interview themes overlapped multiple PCC framework domains (e.g., a participant experience illustrated both Individualized Care and Shared Decision-Making, i.e., changes in medication dose or type). These themes were mapped to a single framework domain through team consensus. Of the four PCC domains, perspectives on Therapeutic Alliance were most frequently mentioned, particularly global expressions of satisfaction with MOUD care. The presence or absence of Individualized Care and Shared Decision-Making were the second most discussed PCC domains.

Although the interview guide contained questions pertaining to Holistic Care (e.g.,. inquiry about MOUD care team interfacing with other social services), themes related to Holistic Care were not reported as frequently (see Table 1 for a summary of the PCC framework and what themes and their respective examples are mapped onto each of its domains). Among the 80% of participants who reported history of prior care at other treatment locations on the demographic screener, appreciation of PCC principles at current care settings were often directly contrasted with the absence of it at past treatment locations.

**Table 1:** Patient-Centered Care (PCC) themes

PCC domain	Defining characteristics	Themes and examples	Valence	Frequency of theme
Therapeutic Alliance	Non-judgmental, respectful and accepting	*Satisfaction*		
		• Overall care satisfaction	O	Majority
	Empathy, understanding, warmth, kindness, supportive	• Satisfaction with care team	O	Majority
		*Trust and Support*		
		• Felt validated	O	Majority
		• Staff attitudes towards use/relapse	O	Some
		• Team responsiveness	O	Some
		• Comfort with disclosure	O	Minimal
		*Communication Quality*		
		• Confidentiality and privacy	-	Minimal
		• Recognition of patient achievements	O	Minimal
		• Dysfunctional communication	-	Minimal
		• Inappropriate staff comments	-	Minimal
Individualized Care	Individualized care and treatment planning	*Treatment Modifications*		
	Delivery of treatment accounting to patients needs and preferences	• Appointment flexibility	O	Majority
		• Adapted care to ongoing needs or preferences	O	Some
	Treatment adapted to clients’ barriers and assets	• Clinic protocols for medication access	-	Some
		• Child-friendly clinic	O	Some
		*Telemedicine Preferences*		
		• Flexibility/comfort of telemedicine	+	Some
		• Accountability/connection of in-person interactions	+	Some
		*Care Continuity*		
		• Care transfer organization	O	Some
		• Patient factor driven discontinuation	-	Minimal
Shared Decision Making	Client and provider dialogue to reach a mutual decision	*Medication decisions*		
	Autonomous decision-making	• Dose changes and medication type	O	Some
		*Collaborative care*		
		• Collaborative procedures	-	Some
		• Care team accountability for clinic substance testing mistakes	-	Some
		• Patient preference for provider-directed care	+	Minimal
		• Clinic abstinence expectations	-	Minimal
		*Continuum of care decision making*		
		• Clinic-driven discontinuation	-	Minimal
		• Collaborative care transfer	-	Minimal
Holistic Care	Integration of physical, mental, and psychosocial support with *MOUD* treatment	*Care team supporting non-substances needs*		
		• Biopsychosocial care coordination	+	Some
		• Team support above and beyond standard care	+	Minimal
	Gender-responsive approach to delivery of treatment	• Parental support	+	Minimal
		*Gender-responsive care*		
	Integration of *MOUD* treatment as part of primary care or hospital setting for other psychosocial needs	• Dose changes related to pregnancy	O	Minimal
		• Education about MOUD and pregnancy/sexual health	-	Minimal
		• Preference of counselor gender	-	Minimal
		*Support for other substance use goals*		
		• Support for problem alcohol use	+	Minimal
		• Support for problem tobacco use	+	Minimal

*PCC Domains and Defining Characteristics reproduced verbatim from Marchand et al., 2019

**Valence was indicated as “+”, “–”, or “O” indicating participant appraisal of a desirable aspect of patient-centered care (+), an undesirable lack of patient-centered care (–), or experiences of both desirable and undesirable aspects of patient-centered care (O). Pervasiveness was denoted in three categories for analytic coherence, a majority (appearing in more than half of the interviews), some (2 to half of the interviews), minimal (1–2 interviews)

### Therapeutic alliance

Themes related to Therapeutic Alliance were *Satisfaction*,* Trust and Support*, and *Communication Quality.* Examples of *Satisfaction* included Overall Care Satisfaction (O) and Satisfaction with the Care Team (O), both of which had reports of mixed experiences across participants. Specifically, all 15 participants discussed their different levels of satisfaction with their care as a whole or with their care team. Positive mentions of overall care satisfaction included sentiments like:

“They’ve just been super nice and helpful… I couldn’t ask for a better experience” (P12; +).

The global expressions of dissatisfaction, mostly describing the lack of Satisfaction with Care Team in previous treatment settings, included statements such as:

“I just didn’t like the experience with the doctors, and how they acted, and treated you” (P10; -).

“I just feel like a lot of people there just don’t do their jobs’’ (P4; -).

The second theme, *Trust and Support*, had mentions of Team Responsiveness (O), Feeling Validated (O), Staff Attitudes Towards Use/Relapse (O), and Comfort with Disclosure (O). Most participants who reported feeling validated by current care team members gave examples of instances where they were trusted by providers, and felt heard, respected, and supported:

“It was scary as hell, but when I got there, the people there were really nice and it was kind of nice to be in a more judgment-free atmosphere. And they listened to what you had to say, told me about the program, what I would need to do, and I gave it a shot” (P13; +).

However, some participants expressed feeling invalidated by staff at clinics attended in the past:

“If I had to keep going with certain doctors and nurses that had the attitudes that I dealt with eight years ago, I wouldn’t be here. That’s for darn sure” (P3; -).

Attitudes about use recurrence from care team members were noticed by patients. One participant shared an experience of staff being “mad” about their return to use (P8, -), but non-judgmental staff attitudes towards recurrence appeared to contribute to Therapeutic Alliance for some participants:

“I told them that because it was about 10 days ago, somebody gave me a hit of crack and I was honest about it. I told them about it and everything and they were just like, well, thank you for honesty…And so it is cool in that way that they didn’t judge me or they weren’t upset or anything with me or anything like that” (P5; +).

Care teams actively responding and attending to patient needs strengthened the alliance between patient and provider:

“I know that if I need anything, it doesn’t matter, I don’t have to wait the month to check in. I can send an email and within an hour I’ll get, they’ll either call me or email me back. It’s just amazing.” (P11; +).

However, inattention from the care team was also indicated by some, and in one case, a patient suggested that a clinic’s financial motivation could be a catalyst for the lack of attention:

“You’re giving us [a] really high dose of something, and you’re not really monitoring it. It’s like we come in, and we’re a money sign. We come in, we get our dose. That’s $150 to the clinic. We walk out the door” (P4; -).

An additional theme of Therapeutic Alliance was *Communication Quality*. Although the coding of relevant text segments substantiating this theme was infrequent, notable patterns emerged referencing Confidentiality and Privacy (-), Recognition of Patient Achievements (O), Dysfunctional Communication (-), and Inappropriate Staff Comments (-). Examples include clinic front desk staff discussing patients’ personal information aloud in the waiting room, patients’ success in treatment being recognized or ignored, a patient responding that he was not interested in quitting smoking and the provider reporting in their note that he refused to quit, and clinic staff discussing with patients their own plans to consume alcohol.

### Individualized care

Themes related to Individualized Care included *Treatment Modifications*, *Telemedicine Preferences*, and *Care Continuity*. Examples of *Treatment Modifications* included Appointment Flexibility (O), Adapted Care to Ongoing Needs or Preferences (O), Clinic Protocols for Medication Access (-), and Child-Friendly Clinic (O). Specifically, the importance of appointment flexibility to account for individual needs was strongly substantiated by a majority. The inflexibility of dosing windows for methadone was prominent among those participants with OTP experience. One participant who spoke on the inflexibility of appointments reported that their fear of missing a dosing window was so strong it led to a severe motor vehicle accident while driving to the clinic:

“I was actually going to [OTP Name] every day to get my daily dose of Suboxone at this point. And we happened to be at my parents’ house, which is a half hour, 45 minutes away. And I woke up late in the morning and the clinic was about to close and I’m like, crap, I’m not going to get my medicine. So I got in the car and I was speeding and I ended up hitting a tree. So when this happened, my whole mindset was, I’m done. I can’t go to this clinic anymore. Look, I almost just died trying to race to the clinic to get my medication. And then my accident was severe and my [redacted] was fractured in multiple places. So I was instantly put into the hospital and put on heavy narcotics again” (P11; -).

For some, their treatment being adapted to their individual needs or preferences included choice of individual or group treatment settings, or freedom to bring their children to visits. While some patients reported negative experiences and frustrations, here is one example of a positive experience:

“She made an exception, and we could do a one-on-one group instead of doing the new person’s group because I have [my children]. I thought that was really awesome that they’re willing to work with me…I really appreciated that because they could’ve just said, ‘Well you have to figure it out’” (P9; +).

The second theme, *Telemedicine Preferences*, contained reports of Flexibility/Comfort of Telemedicine (+) and Accountability/Connection of In-Person Interactions (+), both themes appreciated by an equal number of patients.

“This summer I was supposed to have an appointment, and I was away, and I was able to do a telemedicine for that appointment. That was nice. I didn’t have to come back from my trip early or cut it short or anything” (P12; +).

“There’s just something about being in-person where you’re kind of more, through body language and facial expressions and everything. It’s just kind of a more intimate process…And also for me, one really important component is, it’s just really good for me to have reasons to get out of the house and be around people because I’m very isolated. I live alone and have no social life at this point and I’m very inactive.” (P15; +).

*Care Continuity*, the third theme identified in Individualized Care, consisted of Care Transfer Organization (O) and Patient Factor Driven Discontinuation (-). A few participants expressed feeling ready to transfer to a lower level of care, and in one instance, a patient’s own frustrations with the expectations became an internal factor that led to them discontinuing care altogether:

“I was at a point where I was ready to get take-homes. And then I did everything that they had asked me to, except I was still drinking. And then the last day when I was getting my letter ready to get take-homes, they said that they wanted to give me a test to check for alcohol. I was so fed up and angry, I laughed and I never went back” (P2, -).

Some participants shared their experiences with transferring from one clinic to another. When participants felt that their level of care was meeting their individual needs and that their Care Transfer Organization was effective and efficient, they reported success:

“Yeah, and then eventually they just slowly…gave me a whole month’s worth at a time, and then once I was doing really good and had my clean UAs, they were finally like, ‘Okay, you need to go somewhere, you can just go to your primary.’ And they set me up” (P8; +).

However, the same participant noted a different experience within the same theme of Care Transfer Organization. They felt that communication from their previous providers stopped when they transferred from one care center to another while medication requests were ignored, indicating a pivotal lapse in their individualized care:

“They were supposed to give me a bridge to get me to a doctor, and then I ended up relapsing because there was no communication and the doctor there was like, ‘No, I’m not going to give you the bridge,’ and they just didn’t give me any medication when I left. And so I relapsed…” (P8, -).

### Shared decision-making

Themes related to Shared Decision-Making included *Medication Decisions*, *Collaborative Care*,* and Shared Decision Making*. The main theme of *Medication Decisions* included Dose Changes and Medication Type (O). Participants were evenly split between reports of collaborative patient-provider decisions regarding medication dose adjustments, medication type, and buprenorphine formulation preference, and non-collaborative, provider-directed changes where patients felt their opinions were not accounted for.

“I can tell that they care. They actually listen to me. I was on 12 milligrams of Suboxone but because of the stress and stuff, I called them up and I said I thought I need my dose increased…they’re really nice, really good people” (P5; +).

“When I first went through I was on like 8 milligrams. And I had to go from 8 down to 2 within the course of like two months… the jump from 4 to 2, I was actually still sick. Because I was just dropping down so quickly because the doctor had more or less said, ‘You need to get out’” (P14; -).

*Collaborative Care* had examples of Collaborative Procedures (-), Care Team Accountability for Clinic Substance Testing Mistakes (-), Patient Preference for Provider-Directed Care (+), and Clinic Abstinence Expectations (-). Some participants mentioned clinic procedures such as urine testing and security protocols being inherently uncollaborative. In one instance, a patient recounted a time where they were not given their take home medication when they were unable to provide an observed urine sample due to “stage fright”:

“You can walk in at one point you have a UA and unfortunately for me I had trouble going to [the] bathroom when people were staring at me. I wasn’t used to it and [it] took me years to get through it… And that’s just one night [without medication], because you know you’re going to be sick” (P3, -).

The belief that providers monitoring care could help prevent recurrence, as well as inconsistent expectations for abstinence across clinic staff, and substance testing errors with faulty equipment and mislabeling samples were mentioned minimally.

Coding of relevant text segments substantiating the third theme, *Shared Decision Making*, was infrequent; however, notable patterns emerged referencing Clinic-Driven Discontinuation (-) and Collaborative Care Transfer (-). The reasons cited for clinic-driven discontinuation were use of alcohol or cannabis, missed appointments, pregnancy, or providers independently determining patient level of care requirements and prompting a transfer of care. Unilateral decisions to change MOUD level of care by the providers indicated the complete absence of shared decision making:

“I had kind of graduated out of [the hub], however I did not have good enough insurance to cover my Suboxone at that time. So I had asked the doctor who was running the clinic at the time, to allow me to stay at the clinic. And what he had told me was no, because there were other people who needed that level of care…. I explained to him during the time too like, “I still need this. I’m not stable enough to just go cold turkey, off everything.” But he did not feel the same way I did at that time I had left the clinic. I had relapsed shortly thereafter, probably for about a year” (P14, -).

### Holistic care

Themes related to Holistic Care were identified as *Care Team Supporting Non-Substance Needs*,* Gender Responsive Care*, and *Support for Other Substance Use Goals*. The main examples identified within *Care Team Supporting Non-Substance Needs* were Biopsychosocial Care Coordination (+), Team Support Above and Beyond Standard Care (+), and Parental Support (+). Appreciation for Holistic Care coordination beyond SUD needs was voiced by some participants including care team support for housing, employment, non-substance related mental health needs, clothing, and child custody advocacy:

“She called places with me. Helped me get referrals when I was fighting to have visits with my daughter. She was really helpful with all that stuff, if I needed, with a referral letter. Just anybody that advocates for me; I feel like I had somebody in my corner” (P9; +).

*Gender Responsive Care*, although referenced minimally by participants, had examples in Dose Changes Related to Pregnancy (O), Education about MOUD and Pregnancy/Sexual Health (-), and Preferences of Counselor Gender (-). Patients reported not being informed until the end of their pregnancies about the effect that MOUD could have on their babies. They felt fear and guilt for their infants possibly having side effects, including withdrawals. Additionally, there was a report of a patient requesting a counselor of a specific gender that was originally guaranteed by clinics, but not respected in practice.

The third theme, *Support for Other Substance Use Goals* had mentions of Support for Problem Alcohol Use (+) and Support for Problem Tobacco Use (+). There was minimal reference to care team support for tobacco and alcohol use disorders and no mention of support for co-occurring stimulant or sedative use, despite a majority of participants recounting a history of problematic use of multiple substances including stimulants and sedatives. Participants were not asked directly about treatment for SUDs other than OUD in their interviews with researchers, but many openly shared their histories of use and/or treatment of other substances.

## Discussion

This study examined patient perspectives of their MOUD treatment across Vermont clinics to identify indicators of success and potential challenges patients have experienced. The unique study contribution is the utilization of the PCC framework (a model of care that is associated with increased care utilization for substance use disorders), that was used to guide the organization and interpretation of patient feedback.

Four noteworthy themes of treatment experiences emerged across the PCC domains by a majority of respondents: overall care satisfaction (Therapeutic Alliance), satisfaction with the care team (Therapeutic Alliance), feeling validated by their provided care (Therapeutic Alliance), and appointment flexibility (Individualized Care). Participant experiences with current MOUD care were largely indicative of patient-centered care while the inverse was observed in past episodes of care.

Within the Therapeutic Alliance domain, participants reported that they felt more engaged in their care if it was delivered by attentive, responsive, and non-judgmental care teams. Additionally, participants reported a stronger therapeutic alliance when providers and staff lead with empathy and respect as it pertains to relapse. However, for some patients, they expressed negative experiences when it comes to the quality of communication from staff and providers, leading to a fracturing of their alliance. Therapeutic alliance has repeatedly been positively associated with retention in SUD treatment [29, 30], and retention in MOUD has been strongly associated with improved outcomes and reduced mortality [3, 31].

Regarding Individualized Care, participants endorsed preference for care tailored to their specific needs. Consistent with the literature, Individualized Care themes frequently manifested as desire for MOUD appointment flexibility [32] whereas inflexible scheduling prompted fear of not accessing medication. From previous research we know that a lack of MOUD treatment continuity is strongly associated with relapse and overdose [3, 33], further emphasizing a patient’s desire for flexibility and individualized care considerations. Participant emphasis on appointment flexibility dovetails with the importance of uninterrupted MOUD delivery to treatment outcomes and contributes to the literature suggesting that the constraints of OTP regulation may undermine treatment goals [34]. Furthermore, while some participants reported that they preferred in-person treatment, others valued the option of telemedicine, a delivery modality that supports access to and retention in MOUD [35–37], for increased flexibility in managing their OUD treatment. However, patients’ thoughts on the use of telemedicine in their treatment varied when it was reported (which was minimal). This may indicate that though the possibilities of telemedicine could positively impact treatment in rural areas, more data are needed.

The relationship between Shared Decision-Making and continuing engagement in MOUD at a given clinic was expressed by some participants who shared that they were less likely to continue care when they do not have input into medication and treatment decisions. The enthusiasm participants expressed for being able to collaborate with care teams about medication options and treatment expectations was indicative of engagement in care and suggestive of a relationship between shared MOUD treatment decision-making and retention. This is consistent with previous literature that shared decision-making denotes an underlying philosophy of respect towards clients as integral rather than passive partners in the treatment process. This respect between provider and patient allows for patients to take an active role in their treatment decisions including needs and expectations. The integration of shared decision making in medical care also must pull in the other PCC domains such as Individualized Care, where the patient is bringing their individual needs into the conversation for the shared decision making process to occur [19].

Participants’ reports that holistic assistance with quality of life and relational goals improves connection to care teams, and is consistent with research on patient perception of OUD nurse care managers [38] and perspectives from pregnant and parenting women receiving MOUD [39]. Previous research has also shown strong associations between medical, educational, and mental health services and substance use treatment retention. Improvement of post treatment outcomes has also been noted when treatment consists of wrap-around services such as basic needs, childcare, and family [40]. While support for Holistic Care emerged less prominently than other PCC domains in the data, the Holistic Care themes that did emerge support the importance of biopsychosocial care. Additionally, participants highlighted positive experiences and appreciation when they had parental support from the clinic staff, particularly when they felt that the support given went above and beyond standard care. Examples of this include assistance with housing, employment, non-substance related mental health needs, clothing, and child custody advocacy. Support regarding other substance use goals, such as alcohol, were also appreciated by patients. When holistic care was present for individuals in their treatment, they were supported in several facets of their life, all of which had an impact on their substance use treatment experience.

While much of the PCC framework was mapped, interviewees also discussed challenges faced by rural populations in general (e.g., transportation [41–43], childcare [44]) and legislation that inhibits patient-centered care (e.g., OTP restrictions [45, 46]). Additionally, drug testing procedures vary throughout the State but are required as part of best practice; however, testing is inconvenient and can often lead to mistrust that wears on the Therapeutic Alliance and can contribute to issues with retention [47, 48]. Though patient-centered care is encouraged, addiction treatment in the US is still largely siloed; even in Vermont, which has embraced and supported MOUD at the State level, systemic structures of the addiction care system inherently create barriers to patient-centered care.

### Limitations

Insufficient access to culturally relevant and appropriate SUD treatment including care provided in a patient’s native language has been identified as a treatment barrier [49]. Due to the sample profile of all White English-speaking individuals, this precluded exploration of perspectives informed by experience of receiving MOUD care delivered in an unfamiliar language and cultural context. All study participants received care in Vermont, a state where 94% of the population identifies as White [50]. The lack of racial or ethnic diversity among participants precluded exploration of perspectives informed by experience of racial or ethnic discrimination in health care. Additionally, participants may have been more stable and therefore more likely to have had a positive experience in MOUD given their choice to participate in these interviews, and these may not be generalizable to the experiences of MOUD patients more broadly. Future research should explore patient-centered perspectives at different stages of recovery, different durations in treatment, a culturally and linguistically diverse patient population, and include both housed and unhoused patients.

## Conclusions

To our knowledge, this research is the first primary analysis utilizing the four domains of the PCC framework to understand patient perspectives of patient-centered outpatient MOUD care. Findings support the importance of patient-centered care to individuals receiving MOUD and suggest that patient engagement in care may be superior when clinics attend to fostering a therapeutic alliance, tailor care to individual needs particularly when scheduling visits, incorporate patient preferences into medication and other care decisions, and provide biopsychosocial care or care referrals. Future qualitative exploration into patient centered MOUD care at settings serving culturally and linguistically diverse populations at different stages of care could add to the range and richness of patient perspectives on MOUD treatment delivery.

## Data Availability

No datasets were generated or analysed during the current study.

## Declarations

### Authors’ Information

EGH conducted this research study, data cleaning, analyses, and manuscript writing while at Dartmouth College.
